# Journey to the center of the protein: allostery from multitemperature multiconformer X-ray crystallography

**DOI:** 10.1107/S2059798318017941

**Published:** 2019-01-28

**Authors:** Daniel A. Keedy

**Affiliations:** a Structural Biology Initiative, CUNY Advanced Science Research Center, New York, USA; b Department of Chemistry and Biochemistry, City College of New York, New York, USA; c PhD Programs in Chemistry and Biochemistry, The Graduate Center of the City University of New York, New York, USA

**Keywords:** multitemperature crystallography, multiconformer modeling, protein flexibility, conformational heterogeneity, allostery

## Abstract

Proteins inherently fluctuate between conformations to perform functions in the cell. For example, they sample product-binding, transition-state-stabilizing and product-release states during catalysis, and they integrate signals from remote regions of the structure for allosteric regulation. However, there is a lack of understanding of how these dynamic processes occur at the basic atomic level. This gap can be at least partially addressed by combining variable-temperature (instead of traditional cryogenic temperature) X-ray crystallography with algorithms for modeling alternative conformations based on electron-density maps, in an approach called multitemperature multiconformer X-ray crystallo­graphy (MMX). Here, the use of MMX to reveal alternative conformations at different sites in a protein structure and to estimate the degree of energetic coupling between them is discussed. These insights can suggest testable hypotheses about allosteric mechanisms. Temperature is an easily manipulated experimental parameter, so the MMX approach is widely applicable to any protein that yields well diffracting crystals. Moreover, the general principles of MMX are extensible to other perturbations such as pH, pressure, ligand concentration *etc*. Future work will explore strategies for leveraging X-ray data across such perturbation series to more quantitatively measure how different parts of a protein structure are coupled to each other, and the consequences thereof for allostery and other aspects of protein function.

## Abstract 2

Crystallography at multiple temperatures can reveal the collective shifts of alternative conformations that underlie allosteric communication through protein structures.

## 1. Introduction   

**Figure 1:**
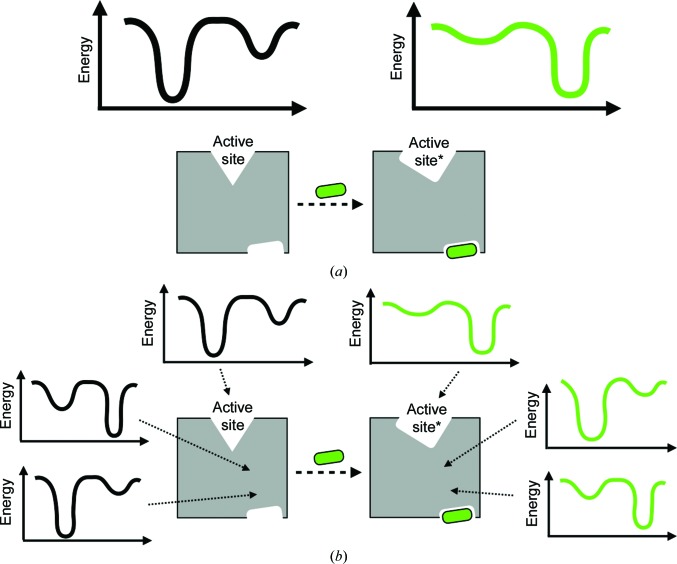
Protein energy landscapes and allostery. (*a*) A generic framework for allostery is that the global energy landscape of the protein is altered by an allosteric effector. Here, the energy landscape is schematized as a plot of free energy versus an arbitrary collective conformational coordinate. An allosteric effector, here a small-molecule ligand binding at an allosteric site, modulates the energy landscape, which changes the conformation of the active site (*), thus altering the function of the protein. (*b*) However, the portrait in (*a*) is agnostic to the mechanisms by which the local energy landscapes of specific regions of a protein structure respond to the allosteric effector and to each other. It therefore remains unclear how the allosteric signal propagates from the allosteric site through the tertiary structure to the functional site. Although this propagation may be branching rather than linear as depicted schematically here, it must ultimately have a physical mechanistic basis that can be understood in structural terms.

Life at the molecular level is fundamentally dynamic. Proteins, the molecular workhorses of cells, are not static entities: rather, they fluctuate between alternative conformations defined by a complex energy landscape (Frauenfelder *et al.*, 1991 ▸) to accomplish their biological functions. One feature of proteins that critically depends on multiple conformations is allostery, the process by which a chemical or molecular signal is transmitted from one site in a protein to another to alter its structure and/or dynamics and therefore its function. Although classical models of allostery have revolved around oligomeric proteins such as hemoglobin, allostery is now recognized as an inherent property of essentially all proteins (Gunasekaran *et al.*, 2004 ▸; Clarkson *et al.*, 2006 ▸). This is fundamentally because protein tertiary structure is complex and interdependent, such that a localized perturbation can shift collective, global degrees of freedom (Fig. 1 ▸ *a*). However, it remains unclear how to decipher which local regions of a protein structure are conformationally coupled to each other in this way, much less the physical basis underlying such coupling (Fig. 1 ▸ *b*). The difficulty of mapping conformational coupling in protein structures poses a major barrier to elucidating the allosteric mechanisms that play key regulatory roles in cellular signaling networks. It further prevents us from rationally exploiting allostery to modulate dysregulated cellular signaling processes for therapeutic purposes.

X-ray crystallography has traditionally been viewed as a static enterprise, with limited applicability to phenomena such as allostery which fundamentally rely on multiple conformational states and the transitions between them. However, owing to the convergence of a number of recent experimental and technical developments, X-ray crystallography is increasingly well positioned to provide insights into the connection between protein flexibility and function. A unifying theme of these advances is an emphasis on multiple conformations and multiple data sets, as emphasized by the theme of the 2018 CCP4 Study Weekend: ‘Multi and Serial Crystal Data Collection and Processing’. These advances are first briefly mentioned here, but are expanded upon in more detail in Section 2.

**Figure 2:**
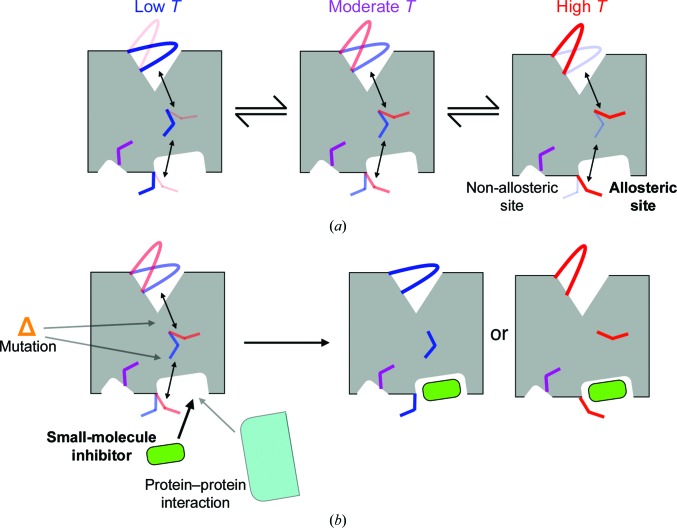
Multitemperature multiconformer X-ray crystallography (MMX) for predicting allostery in protein structures. (*a*) MMX provides a way to infer how local regions of a protein structure mechanically couple to each other to facilitate allostery, as illustrated here schematically for a dynamic enzyme. At low temperature (*e.g.* 100 K), the active-site loop (top) and several residues linking the active site to a distal allosteric site (middle to bottom right) adopt a particular alternative conformation (blue) with higher probability or occupancy (thicker lines). As the temperature is increased (*e.g.* to >200 K), all of these regions concertedly shift their conformational ensemble to include increased populations of a different alternative conformation (red). This coupled behavior does not definitively prove, but is consistent with, the hypothesis that these regions are energetically coupled to each other and thereby form part of an interdependent allosteric network. By contrast, a different residue (bottom left) remains in a single conformation (purple) that is independent of temperature and thus is unresponsive to the other allosterically linked regions. The bottom-right binding site is therefore more likely to be capable of allosteric signaling to the active site than is the bottom-left binding site. (*b*) A molecular perturbation such as a small molecule (green) can test the hypothesis that different parts of the allosteric network are energetically coupled and that biasing the conformation of one part of the network biases the conformations of other parts. Artificial small molecules may compete with natural protein–protein interactions that play regulatory roles in cells (cyan). In addition, mutations (orange) may interfere with the energetic coupling between residues within the network. Thus, these other types of perturbations may equally well be used to interrogate allosteric networks that are predicted using MMX-based approaches.

Firstly (see Section 2.1), new algorithms are emerging to detect and model ‘hidden’ alternative conformations (Lang *et al.*, 2010 ▸; Fig. 2 ▸ *a*; red versus blue states) that are evident in electron-density maps. Different model types have emerged to represent this conformational heterogeneity, including independent multicopy (DePristo *et al.*, 2003 ▸; Terwilliger *et al.*, 2007 ▸), constrained multicopy (Levin *et al.*, 2007 ▸), time-resolved ensemble (Burnley *et al.*, 2012 ▸) and multiconformer (Keedy, Fraser *et al.*, 2015 ▸) models. Some newer methods capture heterogeneity not only for the protein but also for flexible (van Zundert *et al.*, 2018 ▸) or low-occupancy (Pearce, Krojer, Bradley *et al.*, 2017 ▸; Pearce, Krojer & von Delft, 2017 ▸) bound ligands.

Secondly (see Section 2.2), >90% of protein crystal structures have been solved at temperatures below 160 K (M. Gerstel & E. Garman, unpublished observations). However, at ‘room temperature’ (∼277 K) over one third of residues in protein crystal structures feature different (usually broader) conformational ensembles (Fraser *et al.*, 2011 ▸; Keedy *et al.*, 2014 ▸). X-ray data sets collected at intermediate temperatures (Fig. 2 ▸ *a*; left to right) can clarify how conformational coupling relates to biological function, for example at catalytic sites (Keedy, Kenner *et al.*, 2015 ▸) and in allosteric networks (Keedy *et al.*, 2018 ▸). These studies are enabled by the continuing development of strategies for collecting complete, radiation-damage-free X-ray data sets at noncryogenic temperatures (see Section 2.3).

Thirdly (see Section 2.4), X-ray free-electron lasers (XFELs) are revolutionizing crystallography by allowing data collection from microcrystals (Hunter *et al.*, 2014 ▸) and room-temperature data collection without concerns about radiation damage (Hirata *et al.*, 2014 ▸; Keedy, Kenner *et al.*, 2015 ▸; Thomaston *et al.*, 2017 ▸). The ultrafast (femtosecond) timescale and extreme brightness of XFEL pulses also enable the collection of series of data sets related by time delays, offering insights into phenomena such as catalysis and allostery. Many time-resolved XFEL studies use photoactivatable proteins (Tenboer *et al.*, 2014 ▸), but recent (Hekstra *et al.*, 2016 ▸) and near-future developments will vastly broaden the scope of these experiments. Additionally, many XFEL-related developments are trickling down to synchrotrons (Meents *et al.*, 2017 ▸), which will broaden their applicability in the coming years, when synchrotrons will still outnumber XFELs.

Overall, X-ray crystallography is enjoying a renaissance for visualizing conformational ensembles in proteins and how they change during important biological processes. Past reports have emphasized solvent and ice behavior in crystals upon temperature change, contrasting different cryogenic temperatures, and strategies for kinetically trapping specific functional states using temperature (Weik & Colletier, 2010 ▸). Here, I focus on the additional conformations that proteins populate at elevated temperatures, new methods for modeling them and their collective shifts, and prospects for leveraging these models across series of variable-temperature X-ray data sets to map allosteric networks within protein structures. Key concepts include exploiting a series of data sets (rather than just one or two) to build models containing all populated conformations at different occupancies, locally superimposing portions of models to account for conformational changes and non-isomorphisms, and quantitatively comparing maps in real space using those superpositions. The overall approach, which is called multitemperature multiconformer crystallography (MMX), can be used to generate new hypotheses about how molecular signals are energetically transmitted through protein structures to regulate biological function. Such hypotheses can be tested experimentally by imposing chemical or genetic perturbations at different locations in the putative allosteric network, such as small molecules or mutations, and monitoring the structural and functional effects *in vitro* or *in vivo* (Fig. 2 ▸ *b*).

## 2. Advances in methods for detecting allostery   

### 2.1. Modeling multiple conformations in protein structures   

**Figure 3:**
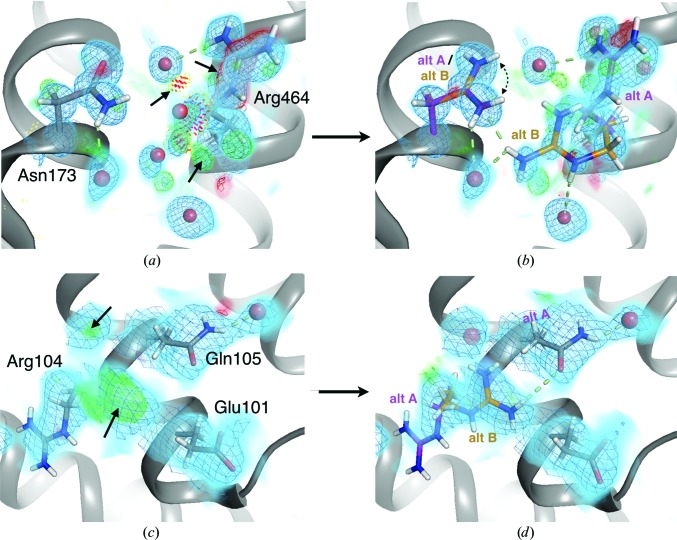
‘Hidden’ alternative conformations in natural and artificial protein structures. (*a*) Natural protein: residues Asn173 and Arg464 in a 0.88 Å resolution structure of catalase (PDB entry 1gwe; Murshudov *et al.*, 2002 ▸) are each modeled with a single conformation. However, 2*F*_o_ − *F*_c_ (0.7σ as a light blue volume and blue mesh) and ±*F*_o_ − *F*_c_ (+3.5σ and −3.5σ in green and red, respectively; both volume and mesh) electron-density maps suggest that the existing Arg464 conformation is overmodeled and reveal evidence for a ‘hidden’ alternative conformation. Supporting this interpretation, the existing Arg464 conformation sterically clashes (red/orange/yellow spikes; Word, Lovell, LaBean *et al.*, 1999 ▸) with several waters (red spheres) that were mistakenly modeled into that electron density. (*b*) A refitted and rerefined model, with the Asn173 side-chain amide flipped 180° (curved dotted arrow; Word, Lovell, Richardson *et al.*, 1999 ▸), an alternative rotamer added for Arg464 (purple versus orange), the offending waters removed and alternative water positions that are mutually exclusive with the original Arg464 conformation added, results in a better fit to the electron density, including diminished *F*_o_ − *F*_c_ difference peaks, elimination of steric clashes and a more extensive hydrogen-bonding network (green dotted lines). Some additional partial-occupancy waters may also be present, given the remaining positive *F*_o_ − *F*_c_ density. (*c*) Artificial protein: residues Arg104 and Gln105 in chain *B* of a 2.09 Å resolution structure of a *de novo* designed protein (PDB entry 5e6g; Jacobs *et al.*, 2016 ▸) are modeled with single conformations. However, 2*F*_o_ − *F*_c_ (0.7σ as a light blue volume and blue mesh) and ± *F*_o_ − *F*_c_ electron-density maps (+3.0σ and −3.0σ in green and red, respectively; both volume and mesh) reveal evidence for a ‘hidden’ alternative conformation for Arg104 and a missing partial-occupancy ordered water molecule nearby that were not specified in the design model (arrows). (*d*) A refitted and rerefined model, with alternative conformations (purple versus orange) for Arg104 and the partial-occupancy water added, results in a better fit to the electron density, including diminished *F*_o_ − *F*_c_ difference peaks and a more extensive hydrogen-bonding network (green dotted lines). Images were obtained using *PyMOL* (Schrödinger).

Proteins populate a variety of conformational states, even in crystals. This idea is supported by the fact that many different single-conformer models explain the diffraction data equally well (DePristo *et al.*, 2004 ▸). *B* factors only model local harmonic disorder and do not account for large-scale motions or alternative conformations (Fig. 3 ▸). The ubiquity of such discrete alternative conformations was driven home by the *Ringer* algorithm, which revealed that over one third of residues in protein crystal structures have enriched electron density at alternative side-chain rotamer positions (Lovell *et al.*, 2000 ▸; Hintze *et al.*, 2016 ▸) in addition to the primary rotamer (Lang *et al.*, 2010 ▸). However, *Ringer* only generates hypotheses about the existence of such alternative conformations. Moreover, it assumes a fixed protein backbone, despite the fact that alternative side-chain conformations are frequently coupled to subtle backbone motions (Davis *et al.*, 2006 ▸; Hallen *et al.*, 2013 ▸). Additional methods are needed to select specific alternative protein conformations, including both side-chain and backbone shifts, in atomic detail.

Recently, several exciting new computational approaches have emerged that harness X-ray diffraction data to automatically model conformational heterogeneity. One recent technique blends crystallographic refinement with simple molecular-dynamics (MD) simulations to construct time-averaged ensembles of a few to dozens of models, each of which is a complete copy of all atoms, that contribute equally to collectively explain the data (Burnley *et al.*, 2012 ▸). In the near future, incorporating more sophisticated force fields such as Amber (Case *et al.*, 2005 ▸) into the MD component of ensemble refinement may significantly improve the quality of the resulting ensembles. Ensemble models are visually appealing and open the door to the statistical inference of correlated motions, for example using information theory (McClendon *et al.*, 2009 ▸), but deriving biological insights from ensemble models remains challenging overall.

In contrast to ensemble models, multiconformer models include multiple positions only for specific atoms where such heterogeneity is sufficiently justified by the data. These alternative conformations are specified by distinct labels (‘A’, ‘B’ *etc*.) and occupancies that sum to unity (or less) per atom. Manually modeling alternative conformations accurately and consistently is difficult because the local electron density is typically weaker than that for well ordered regions of protein structure, and because manual crystallographic modeling in general is subjective and irreproducible. The *qFit* algorithm (van den Bedem *et al.*, 2009 ▸) addresses these challenges by automatically selecting a parsimonious set of 1–4 conformations that collectively explain the local electron density for every residue in a protein structure. Importantly, *qFit* explicitly considers not only side-chain flexibility via rotamers but also backbone flexibility via subtle distributed shifts of backbone atoms in response to side-chain motion. This strategy of coupling side-chain to backbone motions implicitly captures backrub motions, which are subtle dipeptide rotations that are observed in natural proteins (Davis *et al.*, 2006 ▸), as well as more dramatic motions such as peptide flips (Keedy, Fraser *et al.*, 2015 ▸). Although it is common to manually make some minor adjustments to automatically generated *qFit* models, overall *qFit* provides an unbiased route to produce multiconformer models based on X-ray data sets that can be compared across conditions, for example cryogenic versus room temperature or wild type versus mutant.

**Figure 4:**
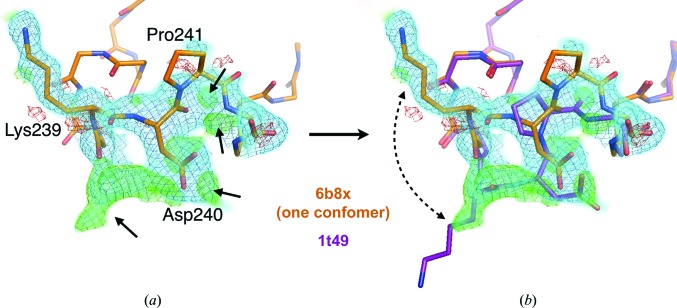
Building multiconformer models by cross-pollinating conformations. (*a*) In a single-conformer version of a multiconformer model for a room-temperature apo (278 K) structure of PTP1B (PDB entry 6b8x; Keedy *et al.*, 2018 ▸), ‘loop 16’ fits the 2*F*_o_ − *F*_c_ (1.25σ as a cyan volume and blue mesh) electron-density map well, but significant positive *F*_o_ − *F*_c_ (+3.0σ and −3.0σ in green and red, respectively; both volume and mesh) peaks remain. It is difficult to visually guess the conformational change that would relate the single-conformer model to the difference density. (*b*) Loop 16 in another structure of PTP1B, at cryogenic temperature with a ligand bound elsewhere (PDB entry 1t49; Wiesmann *et al.*, 2004 ▸), easily explains the difference density, allowing one to combine these states into a multiconformer model (PDB entry 6b8x; Keedy *et al.*, 2018 ▸). Images were obtained using *PyMOL* (Schrödinger).

Although it efficiently models subtle backbone flexibility – for example shifts of 1 Å or less – *qFit* is not equipped to capture larger excursions such as loop motions (Fig. 4 ▸). This gap is significant in light of the fact that protein conformational heterogeneity is often hierarchical (Smith *et al.*, 2015 ▸): for example, the ensemble of alternative conformations of a side chain can depend on larger backbone shifts that are distributed through larger regions of the structure (Deis *et al.*, 2014 ▸). Fortunately, unexplained electron density in such cases is often remarkably well explained by another structure of the same protein or of a similar protein (Best *et al.*, 2006 ▸), as for a loop that is distal from the active site in a room-temperature apo versus a cryogenic inhibitor-bound structure of protein tyrosine phosphatase 1B (PTP1B; Keedy *et al.*, 2018 ▸; Fig. 4 ▸). This may be because the other structure is responsive to a difference in temperature, ligand, sequence or crystal unit cell. In any case, multiconformer modeling methods may benefit in the future from such ‘cross-pollination’ between data sets that are mutually related in some way.

In addition to proteins, methods are emerging to model the conformational heterogeneity of ligands in complex with proteins. Multiconformer models of protein–ligand complexes have the potential to shed new light on entropy/enthalpy trade-offs during binding, intermediate protein–ligand states during functional cycles and the regulatory effects of ligand dynamics on the biological functions of proteins (Srinivasan *et al.*, 2013 ▸). However, alternative conformations for ligands are difficult to detect for several reasons: ligands are less constrained than the polypeptide chain and thus can be more flexible, they may be present only at partial occupancy and their electron density may be obscured by co-located but partially occupied solvent molecules. *qFit-ligand* is a new method that addresses the challenge of identifying multiple ligand conformations by combining a conformational sampling scheme for ligands with the electron-density-based selection algorithm underlying *qFit* for proteins (van Zundert *et al.*, 2018 ▸). In addition, the *PanDDA* algorithm bypasses problems from partial-occupancy solvent by subtracting an estimate of the unbound state of the crystal after real-space electron-density map alignment, resulting in maps that approximate the bound state even for low-occupancy ligands (Pearce, Krojer, Bradley *et al.*, 2017 ▸). *PanDDA* is complementary to ideas such as polder maps, which exclude the bulk-solvent mask from regions of interest (for example ligand-binding sites) during omit-map calculation to more clearly visualize the ligand and/or alternative protein conformations (Liebschner *et al.*, 2017 ▸). These approaches may be productively combined in the future to construct multistate models with the correct combination of mutually exclusive ligand conformation(s) and ordered solvent networks (Pearce, Krojer & von Delft, 2017 ▸).

When evaluating the validity of multiconformer models, it is important to avoid overfitting with respect to the data. In multiconformer models, the number of parameters and thus the parameters-to-observations ratio is multiplied by the number of conformers, but only for those specific atoms with alternative conformations. Generally, the parameters-to-observations ratio for a multiconformer model can be expected to be greater than for a single-conformer model with isotropic *B* factors, but less than for a model with anisotropic *B* factors (Trueblood *et al.*, 1996 ▸) or for an ensemble model with multiple copies of the entire structure (Burnley *et al.*, 2012 ▸). Traditional cross-validation methods such as the free *R* factor, *i.e.* *R*_free_ (Brünger, 1992 ▸), are valuable for avoiding overfitting, but require that a subset of the data be set aside and can themselves be the target of fitting in some senses (Babcock *et al.*, 2018 ▸). New statistical metrics based on information theory (Babcock *et al.*, 2018 ▸) have the potential to provide unique insights into model selection in protein crystallography, especially after future work has clarified the contributions of different types of model restraints towards the parameters-to-observations ratio.

New multistate and multiconformer model types also pose challenges to existing model-validation tools such as *MolProbity* (Williams *et al.*, 2018 ▸), which were originally focused on single-conformer models. Improvements will be needed to ensure both that each global conformation (specified for each atom by ‘A’, ‘B’ *etc*.) is physically realistic and self-consistent, and that the set of conformations in the model is reflective of the experimental data. Additionally, metrics for local model-to-map fit (Tickle, 2012 ▸) will be important for validating models of low-occupancy conformations based on relatively weak electron-density features. Finally, these complex new model types place greater demands on the infrastructure for restraints during model refinement, which will need to be addressed in rigorous ways moving forward.

Once a high-quality multiconformer model has been obtained, what can it tell us about allostery? Some lessons can be learned by examining pathways of ‘falling dominos’ in which individual residues alleviate clashes with their neighbors by switching between alternative conformations (van den Bedem *et al.*, 2013 ▸). However, this approach relies on a simplistic scoring function based on repulsive overlaps of van der Waals radii. Future work could incorporate more sophisticated force fields that include additional physically based terms, both repulsive and attractive. Progress in this direction may also enable tighter integration with approaches using molecular-dynamics simulations to study allostery (Weinkam *et al.*, 2012 ▸; Bowman *et al.*, 2015 ▸). However, this paper discusses an alternative strategy: multitemperature multiconformer crystallography.

### 2.2. Multitemperature crystallography   

A unique approach to mapping allostery in proteins is to use temperature as a proxy for a biological perturbation, and to observe the collective evolution of the conformational ensemble across the structure. The idea behind this approach is that one multiconformer model indicates that the energy landscape has at least a few minima, but a series of multiconformer models at different temperatures reveals the ruggedness of the landscape and possible collective excursions along it. These collective excursions represent coupled conformational motions that may underlie allosteric communication through a protein structure (Fig. 2 ▸ *a*).

Temperature is useful for this approach because it is a fundamental physical property that is easy to manipulate experimentally. In part for these reasons, its effect on protein structure and dynamics was explored using X-ray crystallo­graphy decades ago. For example, in 1979 Frauenfelder and coworkers contrasted four structures of metmyoglobin at 220–330 K and observed that buried versus solvent-exposed residues had different conformational responses to temperature (Frauenfelder *et al.*, 1979 ▸). Their work painted a portrait of metmyoglobin as having an ordered core with semi-liquid surface regions. Later, as the field was embracing cryocrystallography, Tilton and coworkers studied a different protein, ribonuclease A, across a broader temperature range from 98 to 320 K. In addition to confirming that RNase A also has a spatially heterogeneous response to temperature, they reported that the protein expands linearly with increasing temperature and that many atomic *B* factors have a biphasic response to temperature: insensitive at low temperatures and more sensitive at low temperatures (Tilton *et al.*, 1992 ▸).

More recently, multiconformer modeling (see above) was brought to bear on protein crystal structures at different temperatures. Multiconformer analysis revealed that over one third of residues in protein crystals have a different (typically broader) conformational ensemble, including new side-chain rotamer conformations, at room temperature compared with cryogenic temperature (Fraser *et al.*, 2011 ▸). Some of these previously ‘hidden’ protein conformations are critical for biological function (Fraser *et al.*, 2009 ▸).

Broadly speaking, protein conformational ensembles respond to temperature in complex ways. The conformational redistribution upon cryocooling involves a shift from entropically favored states, such as disordered waters and flexible side chains, to enthalpically stabilized states, such as artificially ordered water molecules that hydrogen-bond to side chains that are now more rigid (Keedy *et al.*, 2014 ▸). In addition to altering the equilibrium conformational ensemble, cryocooling crystals also kinetically traps the protein–solvent system in different states (Halle, 2004 ▸). This occurs in an idiosyncratic and irreproducible fashion owing to unavoidable differences in crystal geometry, liquid-nitrogen plunging rate *etc.*, with the result that independent cryogenic structures can be quite different from one another (Keedy *et al.*, 2014 ▸).

Temperature changes are particularly complex at the ‘glass transition’ or ‘dynamical transition’ range around 180–220 K. The nature of this transition has been ‘hotly’ debated (Ringe & Petsko, 2003 ▸), but it is likely to involve a combination (Keedy, Kenner *et al.*, 2015 ▸) of thermal depopulation of protein conformational states (Lee & Wand, 2001 ▸) and solvent-driven arrest of further evolution of protein conformational disorder (Vitkup *et al.*, 2000 ▸). Importantly, the transition is not global and simultaneous; rather, different residues undergo individual local transitions at different temperatures, as encoded by the details of the rugged energy landscape (Tilton *et al.*, 1992 ▸; Keedy, Kenner *et al.*, 2015 ▸). This fact proved useful in revealing that the residues constituting a dynamic active-site network in cyclophilin A (CypA; Fraser *et al.*, 2009 ▸) are only imperfectly coupled to each other (Keedy, Kenner *et al.*, 2015 ▸) and in mapping an expanded allosteric network of mutually conformationally coupled residues, including a new functionally linked allosteric site, in PTP1B (Keedy *et al.*, 2018 ▸).

Because of the complexity of the glass transition, in the future it may be most fruitful to focus on temperatures above the glass transition to reveal allosteric networks with MMX. At such temperatures, conformational redistributions at different locations in the structure can be more readily understood as mutually interacting responses to the thermal (de)population of conformations at other locations, as opposed to being kinetically trapped by glassy solvent at particular solvent-exposed locations.

### 2.3. Data-collection improvements   

To obtain multitemperature series of data sets for MMX, several technical challenges need to be addressed during experimental data collection. Firstly, crystals can rapidly dehydrate when removed from their mother liquor (Farley *et al.*, 2014 ▸). In some cases, purposeful crystal dehydration prior to cryocooling can be a strategy to improve diffraction quality for cryocrystallography (Russi *et al.*, 2011 ▸; Russo Krauss *et al.*, 2012 ▸). However, over longer periods of time at room temperature, dehydration often degrades diffraction quality. This effect can be reduced by fitting a capillary over the loop containing the crystal, with a reservoir of well solution at its tip. Alternatively, the crystal can be transferred to a drop of protective oil, or the oil can be used to cover the drop such that the crystal becomes coated with it as it is removed from the drop (Warkentin & Thorne, 2009 ▸). Options include Paratone, NVH or other oils, which have various viscosities and other properties that are more or less compatible with the handling of different crystals.

Secondly, crystals must be cooled to each desired temperature. For lower temperatures, traditional cryocooling practices lead to distortions of the conformational ensemble that are, moreover, irreproducible (Halle, 2004 ▸; Keedy *et al.*, 2014 ▸). However, such cooling is performed by manually plunging a crystal into a pool of liquid nitrogen with a gas layer on top that creates a temperature gradient over the course of ∼0.1–1 s. During the time the crystal traverses this temperature gradient, structural relaxation processes can occur within the protein on various timescales that trap it in nonphysio­logical conformations (Halle, 2004 ▸). Two attractive alternatives to such intermediate-timescale cooling include very slow or very fast cooling. Very slow cooling (many minutes) with a cryojet ensures that the contracting protein unit cell and the expanding solvent can equilibrate at each temperature (Warkentin & Thorne, 2009 ▸). By contrast, very fast cooling or ‘hyperquenching’ (∼0.01 s) outpaces many structural relaxation processes for the protein and perhaps especially the solvent (Warkentin *et al.*, 2006 ▸). Although hyperquenching may eliminate the need for cryoprotectants, it does not truly capture the room-temperature ensemble: some relatively rapid structural relaxation processes will, in general, still outpace the cooling, and vibrational modes will be suppressed at the lower temperature. For temperatures above the glass transition range (>180–220 K), crystals can be cooled directly by a cryojet pre-set to the desired temperature, either in a capillary or coated in NVH or another oil (Keedy, Kenner *et al.*, 2015 ▸).

Thirdly, X-ray-induced radiation damage is an ever-present danger in crystallography. This is especially true for variable-temperature strategies such as MMX since radiation damage is temperature-sensitive, with cryogenic temperature providing up to an ∼100-fold protection relative to room temperature (Warkentin *et al.*, 2013 ▸). Because of its potentially pernicious effects, it is important both to limit radiation damage during data collection and to carefully monitor for its existence after data collection so that electron-density changes owing to radiation damage are not misinterpreted; both of these aspects are explored below.

To limit radiation damage by spreading the radiation dose over a larger area of the crystal, crystals can be translated along the goniometer axis during data collection in a procedure known as helical data collection owing to the path traced out by the crystal. More generally, the *RADDOSE*-3*D* software can model radiation damage for specific crystal geometries and propose optimal X-ray dosage strategies (Bury *et al.*, 2018 ▸). Limiting radiation damage by adjusting the dosage can be successful: a controlled study of accumulated radiation-damage series for three proteins at cryogenic versus room temperatures, with total damage adjusted based on the temperature dependence of radiation damage, concluded that radiation damage does not account for the increased protein conformational heterogeneity that is observed at room temperature (Russi *et al.*, 2017 ▸). Moreover, because non-instantaneous structural relaxation processes precede the manifestation of radiation damage, faster data-collection rates may partially outrun radiation damage at room temperature and especially at cooler temperatures that are just above the glass transition (Southworth-Davies *et al.*, 2007 ▸). This is particularly feasible at new third-generation synchrotrons with higher flux densities (Warkentin *et al.*, 2013 ▸).

Complementary to limiting radiation damage during data collection, it is prudent to check for its manifestations before any structural analysis that might lead to inferences about biology. The effects of damage are mostly global, but partially local: for example at solvent-exposed instead of buried regions (Warkentin *et al.*, 2012 ▸). Other specific local effects include the decarboxylation of Asp and Glu side chains and the breakage of disulfide bonds. New computational methods are emerging to quantitatively check series of electron-density maps for artifacts of local radiation damage (Bury *et al.*, 2016 ▸). In addition to closely examining the X-ray diffraction data, one should also use complementary methods such as online UV–Vis microspectrophotometry (McGeehan *et al.*, 2009 ▸; Garman, 2010 ▸) to determine whether radiation damage has occurred and thus to avoid making any spurious conclusions based solely on the diffraction data.

Fourthly, recent technical advances have dramatically increased the throughput of crystallography, which presents challenges at the intersection of data-collection strategies and downstream data processing. Automated robotics-driven sample handling and data collection are increasingly the standard at new beamlines (Winter & McAuley, 2011 ▸; Fuchs *et al.*, 2014 ▸; Cohen *et al.*, 2002 ▸; Muchmore *et al.*, 2000 ▸; Cipriani *et al.*, 2006 ▸; Papp *et al.*, 2017 ▸). High-throughput data collection is now more feasible with *in situ* data collection on microfocus beamlines (Axford *et al.*, 2012 ▸; Perrakis *et al.*, 1999 ▸; Cusack *et al.*, 1998 ▸; Yadav *et al.*, 2005 ▸; Bingel-Erlenmeyer *et al.*, 2011 ▸; le Maire *et al.*, 2011 ▸). Such experiments are shifting the bottleneck in crystallography from data collection to data processing. Fortunately, algorithmic advances, such as automatic processing pipelines (Krug *et al.*, 2012 ▸; Winter, 2010 ▸; Incardona *et al.*, 2009 ▸; Monaco *et al.*, 2013 ▸), are also progressing rapidly to help to address this issue. In many cases, high throughput opens the door to structural analyses on un­precedented scales that provide new biological insights: for example, into low-occupancy protein–ligand interactions (Pearce, Krojer, Bradley *et al.*, 2017 ▸).

Although many high-throughput and multi-data-set experiments produce full data sets which each derive from a single crystal (Pearce, Krojer, Bradley *et al.*, 2017 ▸; Keedy, Kenner *et al.*, 2015 ▸), other modern approaches generate many partial data sets from separate crystals, both with XFELs and at synchrotrons. In these cases, non-isomorphism between crystals can complicate downstream analysis. However, new computational methods (Diederichs, 2017 ▸; Foadi *et al.*, 2013 ▸; Giordano *et al.*, 2012 ▸) have made strides in separating partial data sets (or even single diffraction images) into classes to bypass this problem. Polymorphic crystals can even reveal new information about distinct protein conformations (Ebrahim *et al.*, 2019 ▸). For MMX, such approaches for deconvoluting conformational states from multiple crystals may prove to be broadly useful to more fully reveal the conformational ensemble accessible to the protein at each temperature, especially for systems that yield small crystals which are amenable only to serial microcrystallography rather than to the more traditional fixed-target single-crystal approach. In addition, it has been shown that data sets from crystals of the same protein are more similar to each other at room temperature than at cryogenic temperature (Keedy *et al.*, 2014 ▸). Therefore, to distinguish between the non-isomorphism that is inherent between crystals versus non-isomorphism that is owing to temperature change, future MMX experiments may benefit from finely sampling elevated temperatures (>200 K) so that each data set can be compared only with ‘adjacent’ data sets from similar temperatures.

### 2.4. X-ray free-electron lasers   

In addition to the advances in data-collection methodology described above, the advent of X-ray free-electron lasers (XFELs) removes many obstacles in the path towards serial noncryogenic crystallography. This progress is made possible by the availability of new XFEL sources in the United States, Japan and Europe, as well as associated advances in sample delivery (Baxter *et al.*, 2016 ▸; Fuller *et al.*, 2017 ▸; Sierra *et al.*, 2016 ▸; Oberthuer *et al.*, 2017 ▸; Mafuné *et al.*, 2016 ▸; Liu *et al.*, 2014 ▸; Weierstall *et al.*, 2014 ▸) and data processing (Uervirojnangkoorn *et al.*, 2015 ▸; White *et al.*, 2016 ▸; Hattne *et al.*, 2014 ▸; Winter *et al.*, 2018 ▸). XFELs allow the circumvention of cryocooling to ameliorate radiation damage, in that the phenomenon of ‘diffraction before destruction’ enables damage-free room-temperature data collection (Chapman *et al.*, 2011 ▸). For this reason, XFELs could straightforwardly be used for experiments at multiple temperatures in the regime above the glass transition (>180–220 K): for example for nanocrystals to microcrystals that are too small or otherwise not amenable to synchrotrons.

A second key advantage of XFELs is that they enable access to the time dimension. Time-resolved series of data sets (Tenboer *et al.*, 2014 ▸; Kupitz *et al.*, 2014 ▸; Barends *et al.*, 2015 ▸; Pande *et al.*, 2016 ▸; Shimada *et al.*, 2017 ▸) can directly visualize protein motions that may be relevant to function. This method is an attractive alternative to time-resolved Laue crystallo­graphy, which has stringent technical limitations on parameters such as crystal mosaicity. Time-resolved XFEL crystallography has thus far centered around the photoactivation of specific model systems (Tenboer *et al.*, 2014 ▸; Kupitz *et al.*, 2014 ▸; Barends *et al.*, 2015 ▸; Pande *et al.*, 2016 ▸; Shimada *et al.*, 2017 ▸). However, new tools are being added to the time-resolved XFEL toolkit that will move the field beyond this limitation, allowing much wider reaching explorations of how protein structures dynamically respond to perturbations. For example, the ‘mix-and-inject’ strategy takes advantage of the small crystals that can be analyzed with XFELs to rapidly soak in ligands and initiate biochemical reactions in the crystal (Schmidt, 2013 ▸; Stagno *et al.*, 2017 ▸).

One drawback of the mix-and-inject strategy is that it is effectively restricted to a known ligand and its binding site, which limits the ability to characterize the allosteric connectivity of the entire protein. By contrast, rapid electric field pulses exert forces on partial charges at certain atoms in protein structures, which reveals coordinated motions of some residues (Hekstra *et al.*, 2016 ▸). This ‘exciting’ approach is complementary to varying the experimental temperature, as it is also a global perturbation that can reveal mechanically coupled components of a structure. It is precisely these mechanically coupled structural elements that are likely to participate in intramolecular allosteric signaling networks. Both electric fields and variable temperature are valuable in that they are generalizable to any macromolecule that can be crystallized. In addition, new methods are being developed for inducing rapid temperature jumps in protein crystals by laser excitation of the surrounding solvent (Thompson *et al.*, 2018 ▸). Challenges remain in terms of technical execution, as well as modeling the kinetic propagation of incipient thermal energy from surrounding solvent into the protein surface and core. However, such time-resolved temperature-jump approaches promise to provide novel insights into dynamic aspects of many structural processes, including allostery, and thus will be highly complementary to other time-resolved experiments, as well as equilibrium multitemperature comparisons as in MMX. Moreover, these new time-resolved experiments are largely extensible to third-generation synchrotrons. Although the accessible timescales at synchrotrons are generally slower than at XFELs, in the millisecond instead of the femtosecond range, this can be improved to ∼100 ps using a polychromatic ‘pink-beam’ approach (Meents *et al.*, 2017 ▸).

## 3. Discussion and future directions of MMX   

**Figure 5:**
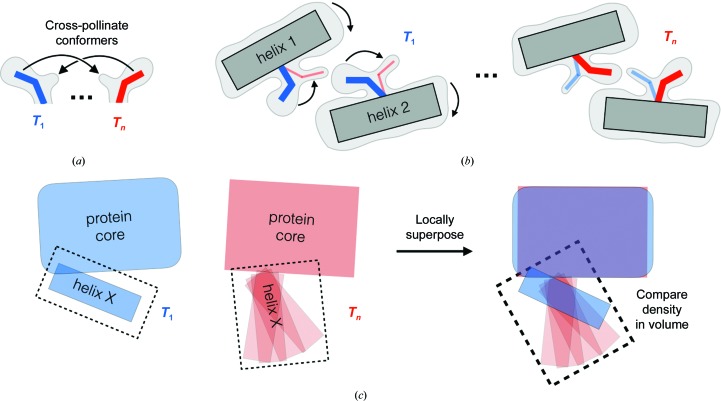
Outline of the intended MMX approach for identifying coupled conformational motions. This manuscript discusses a new paradigm in structural biology: multitemperature multiconformer crystallography (MMX). Future approaches based on MMX will identify residues whose conformational ensembles change concertedly with respect to temperature, which could predict energetically coupled residues that are key to allosteric communication through a protein structure. (*a*) To ensure that future MMX-based approaches compare related data sets in an unbiased way, it will be important to build a sufficiently complete multiconformer model at each temperature. This may be improved by ‘cross-pollinating’ conformers between models at different temperatures. Some of the occupancies of these conformers may refine to low but appreciable values, which will aid in identifying coordinated changes in mixtures of states (Smith *et al.*, 2015 ▸). (*b*) Conformational changes will be monitored by changes in the electron-density map or refined occupancies as a function of temperature. In the schematic example depicted here, the side chains of two residues on adjacent helices in the tertiary structure have mutually exclusive conformations, and the helix–helix interface reconfigures as the populations of the side chains shift from one collective state to another with temperature. Similar analyses could also be performed with other experimental perturbations such as humidity, pH, pressure, ligand concentration *etc.* in future MMX experiments. (*c*) To capture the more complex conformational transitions involving subtle distributed backbone motions that occur in proteins (Deis *et al.*, 2014 ▸), the principles of MMX can be used to superpose maps in real space based on models (Pearce, Krojer, Bradley *et al.*, 2017 ▸) and to examine not just arbitrary volumes of space, but rather structural elements that may move as a cooperative unit – for example, the volume around (dotted rectangle) an α-helix whose conformational ensemble shifts from ordered to quasi-disordered (semi-transparent rectangle), or β-sheets, loops and other ‘fragments’ that compose protein structure (Rohl *et al.*, 2004 ▸).

The MMX approach is well positioned to build on all of these exciting developments in multi-data-set X-ray crystallography to predict allosteric mechanisms in proteins in the future. The idea behind MMX is to build a complete multiconformer model for each data set, locally superpose electron-density maps based on these models (Pearce, Krojer, Bradley *et al.*, 2017 ▸) and quantitatively compare the maps (Fig. 5 ▸). This will reveal which regions or volumes of the protein structure change synchronously. Finally, one will be able to reference from these volumes back to the atomic models to reach biological inferences.

**Figure 6:**
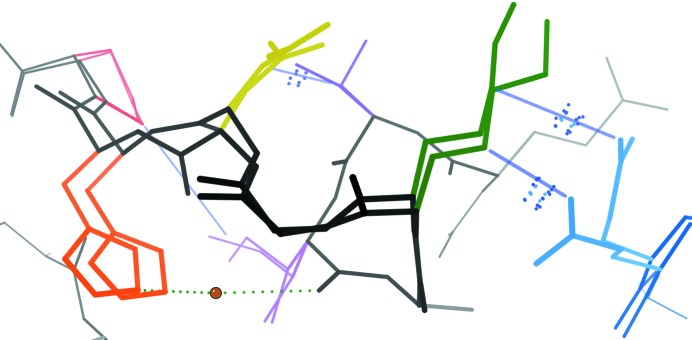
A complex network of coupled conformational heterogeneity. A network of alternative conformations in a cryogenic structure of catalase (PDB entry 1gwe; Murshudov *et al.*, 2002 ▸) with diverse properties. Multiple phenomena define the network: van der Waals interactions (blue dots and line segments) between side chains, a hydrogen bond (dotted green line) through a partial-occupancy water (brown), coupling through the locally mobile backbone (black) and perhaps electrostatic forces between the Lys (green) and nearby polar residues (Glu in blue, Asp in yellow and Ser in purple). This image was obtained using *KiNG* (Chen *et al.*, 2009 ▸).

Synergy between models and maps is a key feature of the proposed MMX paradigm, as focusing on either alone would have limitations. A strictly model-based approach to predicting allosteric mechanisms would be highly sensitive to precise atom placements. Even for simple interaction types such as van der Waals (van den Bedem *et al.*, 2013 ▸), sub-ångström differences can lead to large differences in calculated interaction energies; electrostatics, hydrogen bonds, backbone torsional strain, mobile solvent *etc.* (Fig. 6 ▸) pose additional challenges to force fields. By contrast, a purely map-based approach would bypass some of the limitations of atomic models by more directly interrogating the experimental data. However, superposing multiple maps for comparison is made difficult by non-isomorphism and/or conformational redistributions between the data sets. Moreover, crystallo­graphic maps (usually) derive phases from the model, and thus improvements to maps depend on improvements to models. Overall, MMX-based approaches in the future are likely to benefit from marrying models and maps in a cohesive analysis. Firstly, some maps will more clearly reveal the presence of alternative conformations that are difficult to detect but are nonetheless present in other maps at lower occupancies. To reduce model bias, these conformations can be cross-pollinated across models and their occupancies can then be appropriately refined (Fig. 5 ▸ *a*). Difference refinement (Terwilliger & Berendzen, 1995 ▸) may also be useful for minimizing model bias in this regard. Secondly, the improved maps resulting from these improved models can be compared using local superposition schemes (Pearce, Krojer, Bradley *et al.*, 2017 ▸; Fig. 5 ▸ *c*), thus allowing inferences about conformational changes based on changes in local map values.

As the ideas behind MMX are developed into actual computational methods, a few caveats should be kept in mind. Firstly, coordinated changes in map density and atomic occupancies for contiguous regions of the structure may arise not from biologically relevant energetic coupling, but rather simply from thermally driven repopulation that is site-independent: in other words, correlation rather than causation. This could be addressed by using a large number of data sets to enable ‘fine slicing’ with respect to the perturbation (for example temperature), which could help to reveal whether conformational features are consistently coordinated across the entire perturbation range versus just a portion of it. Additionally, relative analysis of different regions of the structure, including the many regions that do not undergo coordinated changes in density and occupancies, may be useful to reveal the extent to which such generic thermal repopulation occurs. A second caveat is that, as with any crystallo­graphic analysis, lattice contacts may interfere (Tyka *et al.*, 2011 ▸). This may be of particular interest for MMX since lattice contacts rearrange in response to temperature-dependent unit-cell volume changes (Keedy *et al.*, 2014 ▸). This could be partially addressed by considering the distribution of these contacts during the analysis: certainly, any interpretations are subject to further validation if there is a significant direct lattice contact with the region of interest, and one should also be cautious if there are lattice contacts with nearby regions. One could also repeat the analysis in another crystal form with different lattice contacts and ensure that the interpretation of coupled conformational heterogeneity is similar. Note that a crystalline environment does not in general prevent proteins from achieving multiple functionally relevant states, given that some enzymes are indeed active in crystals (Kiefer *et al.*, 1998 ▸). Broadly speaking, MMX will provide an avenue towards hypotheses about conformational coupling between regions of a protein structure, but ultimately experiments are a critical next step.

By treating models and maps synergistically with the MMX approach, one may be able to gain insights into a variety of allosteric mechanisms. In some cases, allostery is dominated by side-chain rotamer changes (Fraser *et al.*, 2009 ▸; van den Bedem *et al.*, 2013 ▸). In other cases, subtler, larger-scale and/or more collective backbone motions are involved (Passner *et al.*, 2000 ▸; Volkman *et al.*, 2001 ▸; Xiao *et al.*, 2004 ▸; Huse & Kuriyan, 2002 ▸). In the future, it will be interesting to use the principles of MMX to examine which classes of backbone motions are involved in allosteric mechanisms: β-sheet flexing, which has been observed across sets of published structures (Fenwick *et al.*, 2014 ▸); side chain–backbone coupling via backrubs (Davis *et al.*, 2006 ▸) or via α-helix shifts (Deis *et al.*, 2014 ▸) *etc.* (Fig. 6 ▸). These examples highlight the challenges involved with inferring allosteric mechanism either purely from models, which may not capture such an eclectic medley of conformational features and interactions, or purely from maps, which would require accurate models for the proper local real-space superpositions to enable map comparisons (Pearce, Krojer, Bradley *et al.*, 2017 ▸).

As computational design of *de novo* proteins (Huang *et al.*, 2016 ▸) continues to mature, it will be promising to contrast natural proteins, in which coupled conformational heterogeneity may serve some functional purpose, with proteins that are designed for unique structure yet contain some vestigial flexibility (Fig. 3 ▸ *a* versus Fig. 3 ▸ *b*). Such comparisons may help to reveal the extent to which coupled conformational heterogeneity is a signature of allostery, catalysis or some other biological function versus simply a consequence of the complex energy landscapes that inevitably arise from a polypeptide defined by a limited amino-acid alphabet. Furthermore, coupled conformational heterogeneity in natural proteins may be able to provide lessons that will aid the future computational design of multistate proteins with new functions (Joh *et al.*, 2014 ▸; Hallen & Donald, 2016 ▸) such as novel allosteric regulatory mechanisms (Taylor *et al.*, 2016 ▸; Khersonsky & Fleishman, 2017 ▸).

Temperature is an easily accessible, general and physically meaningful perturbation. However, importantly, the algorithms from equilibrium MMX can be applied to series of X-ray data sets that are related to each other in some way other than temperature. For example, humidity (Kodandapani *et al.*, 1990 ▸; Sanchez-Weatherby *et al.*, 2009 ▸; Douangamath *et al.*, 2013 ▸), pH (Thomaston *et al.*, 2015 ▸) and pressure (Fourme *et al.*, 2001 ▸; Urayama *et al.*, 2002 ▸) can be varied experimentally. In each case, similar multiconformer modeling and real-space map comparison algorithms could be used to make inferences about coupled conformational changes.

Related algorithms are also emerging in the burgeoning field of cryo-electron microscopy (cryo-EM). For example, a technique called manifold embedding projects micrograph images onto a reduced-dimensionality space, from which 3D reconstructions can be made at various points (Frank & Ourmazd, 2016 ▸). This essentially allows the creation of a series of maps representing continuous conformational changes, which may be biologically relevant. Recently, manifold embedding has been used to elucidate a broad ensemble of trajectories from unbound to ligand-bound states for a receptor protein, most of which involve conformational changes both before and after ligand binding (Dashti *et al.*, 2017 ▸). Another recently reported approach decomposes tertiary or quaternary structures into independent bodies and assesses their relative movements (Nakane *et al.*, 2018 ▸). In the future, it will be fruitful to share computational methods between MMX and cryo-EM for building parsimonious models across series of maps to predict allosteric mechanisms. These methods may also be applicable to lower-resolution X-ray data-set series, broadening the scope of MMX. Furthermore, it may be possible to equilibrate cryo-EM samples to various temperatures before hyperquenching in liquid ethane, allowing comparisons between multitemperature series using cryo-EM and X-ray crystallography.

XFELs also offer many opportunities that are relevant to MMX. Firstly, they offer a radiation-damage-free control on room-temperature data sets from synchrotrons. This approach could also be easily adapted to generate multitemperature series. Secondly, XFELs can generate series of data sets that are related by differential time delays upon any number of perturbations: often light activation (Tenboer *et al.*, 2014 ▸), but also more general methods such as ligand injection (Schmidt, 2013 ▸; Stagno *et al.*, 2017 ▸) or electric field pulses (Hekstra *et al.*, 2016 ▸). Notably, time-resolved perturbation series from XFELs may soon include temperature jumps (Thompson *et al.*, 2018 ▸). Time-resolved XFEL data-set series in general may benefit from algorithms for MMX, which could aid in analyzing how the mixture of states in a protein structure evolves as a function of time in response to a stimulus.

It has been proposed that allostery is driven by changes in dynamics alone in some cases, with no changes in conformation (Cooper & Dryden, 1984 ▸). In support of this view, NMR relaxation experiments have explored dynamically or entropically driven allostery in several protein systems (Wand, 2001 ▸; Popovych *et al.*, 2006 ▸; Petit *et al.*, 2009 ▸). Although it is clear that changes in the rates of conformational dynamics can play important roles in allostery, it seems highly unlikely that the energy barriers between conformations (and thus the rates of dynamics) can change at multiple sites in a complex system such as a protein with zero change to the energies of the conformations themselves (and thus the conformational ensemble of the protein). The use of MMX can help to test the hypothesis that subtle conformational shifts do in fact occur in such systems, but were previously unrecognized because multitemperature and multiconformer X-ray approaches were unavailable. Moreover, time-resolved XFEL experiments, either with currently available perturbations (Hekstra *et al.*, 2016 ▸; Stagno *et al.*, 2017 ▸) or with temperature jumps (Thompson *et al.*, 2018 ▸) in the future, can offer more direct insights into the kinetic aspects of protein allostery.

Overall, MMX has the power to add dimensionality to the X-ray crystallographic analysis of proteins or other macromolecules, which can provide richer insights into the complex energy landscapes that underlie their dynamic functions. It represents an advance from one or two data sets (a point or a line) to many continuously related data sets (a curve). Combining different perturbations such as temperature, pressure, pH *etc.* (Urayama *et al.*, 2002 ▸) will further add to this dimensionality. By using ‘families of models’ to map how different parts of a structure collectively respond to stimuli, MMX has the potential to help to reveal the mechanisms by which information is allosterically communicated through macromolecules.
